# Chronic Inflammatory-Related Disease and Cardiovascular Disease in MESA

**DOI:** 10.1016/j.jacadv.2025.101640

**Published:** 2025-03-03

**Authors:** Evan S. Manning, Gautam R. Shroff, David R. Jacobs, Daniel A. Duprez

**Affiliations:** a Cardiovascular Division, Department of Medicine, School of Medicine, University of Minnesota, Minneapolis, Minnesota, USA; b Division of Cardiology, Department of Medicine, Hennepin Healthcare, Minneapolis, Minnesota, USA; c University of Minnesota Medical School, Minneapolis, Minnesota, USA; d Division of Epidemiology & Community Health, School of Public Health, University of Minnesota, Minneapolis, Minnesota, USA

**Keywords:** chronic inflammation, cardiovascular disease, chronic inflammatory-related disease, inflammation biomarkers

## Abstract

**Background:**

Inflammation plays a role in cardiovascular disease (CVD). We defined various noncardiovascular and noncancer conditions, both infectious and noninfectious, with a common basis of inflammation, collectively termed chronic inflammatory-related disease (ChrIRD). We describe ChrIRD and its interplay with CVD during follow-up in the Multi-Ethnic Study of Atherosclerosis.

**Objectives:**

The aim of the study was to describe ChrIRD, its associations with CVD, and its association with mortality.

**Methods:**

Participants were free of overt CVD at baseline with median 17.9 (Q1-Q3: 14.9-18.6) years of follow-up. ChrIRD was determined by review of hospitalization and death records of International Classification of Diseases codes. CVD diagnosis was adjudicated based on medical records. We performed time-dependent proportional hazard regressions to identify risks related to ChrIRD or CVD events.

**Results:**

MESA (Multi-Ethnic Study of Atherosclerosis) participants (n = 6,791) had a mean age of 62 ± 10 years, with 47% (3,201/6,791) men, 39% (2,617/6,791) White, 28% (1,882/6,791) Black, 22% (1,489/6,791) Hispanic, and 12% (803/6,791) Chinese race/ethnicity. ChrIRD was observed in 29% (1,965/6,791) and CVD in 21% (1,420/6,791); including 11% (761/6,791) with both conditions. Mortality after ChrIRD only was 47% (567/1,204; 95% CI: 44%-49%); after CVD only was 45% (300/659; 95% CI: 41%-49%); and after both conditions was 67% (510/761; 95% CI: 63%-70%). CVD was associated with increased risk of ChrIRD (HR: 1.48, 1.23-1.77) and ChrIRD was associated with increased risk of CVD (HR: 2.23, 1.97-2.52). Baseline inflammatory markers predicted both conditions.

**Conclusions:**

ChrIRD is common, present in all organ systems, and is associated with significant mortality, particularly in combination with CVD. The association between CVD and ChrIRD is bidirectional, and baseline inflammatory markers are associated with ChrIRD and CVD.

Inflammation is a complex interplay of localized immune response and tissue modulation present in a wide range of physiologic and pathologic conditions. When appropriately regulated, inflammation is vital to many physiologic functions such as metabolism and immune defense and, when dysregulated, is implicated in numerous pathologic phenotypes such as obesity, cardiovascular disease (CVD), and cancer as well as severe acute illness states such as cytokine storm syndromes.^1^, ^2^, ^3^, ^4^ The specific role of the inflammatory cascade in many of these conditions is under investigation; however, it is established that low-grade, chronic inflammation, as evidenced by markers such as high-sensitivity C-reactive protein (hs-CRP) and interleukin (IL)-6, is associated with a greater burden of many diseases and increased mortality.^5^ Despite a common pathway of proinflammatory cytokines, leukocyte-inducing factors, and histamine, inflammatory dysregulation has diverse phenotypic expression ranging from autoimmunity and rheumatologic conditions to completely dissimilar clinical phenotypes associated with infection.

Inflammatory dysregulation is increasingly recognized to have a close association with the development and progression of atherosclerotic cardiovascular diseases, likely due to the chronic inflammatory nature of atherosclerosis. However, the clinical relationship between inflammation and CVD is inadequately understood, particularly among individuals with chronic inflammatory conditions.^6^ Insight into the temporal relationship between the 2 disease states, biochemical profiles, associated risk factors for each condition, and overall morbidity and mortality is lacking. A better understanding of the relationship between chronic inflammatory diseases and CVD could improve individual risk stratification, triage for additional cardiovascular testing to detect early CVD, and ultimately improve preventive management of this population, relative to both CVD and other diseases.

Therefore, we studied a composite of a wide variety of noncardiovascular, nondiabetes, and noncancer pathologies, both infectious and noninfectious, with the common basis of inflammation as the fundamental cause; collectively termed chronic inflammatory-related disease (ChrIRD). This clinically defined disease cluster is termed chronic due to an assumption that participants with these conditions have a subclinical predisposition to inflammatory disease, such as a compromised immune defense, propensity for autoimmunity, or an otherwise altered metabolic state that is favorable for inflammation. We examined the demographic, clinical, and inflammatory biomarker distribution of ChrIRD as well as its association with CVD in the MESA (Multi-Ethnic Study of Atherosclerosis) longitudinal cohort who were free of overt CVD and other major, life-threatening conditions at enrollment. We hypothesized that this clinically defined disease cluster has close correlations with CVD, leads to excess death rates, and is correlated with baseline levels of several biomarkers of inflammation.

## Methods

### Study sample

The MESA cohort was initiated to investigate the prevalence and progression of subclinical CVD among individuals initially free of overt clinical CVD at enrollment. Between 2000 and 2002, 6,814 men and women of White, Black, Hispanic, or Chinese race/ethnicity, age 45 to 84 years, were recruited from 6 communities across the United States.^7^ The 6,791 participants with any follow-up data were included in these analyses. By design, MESA participants were nonpregnant and free of clinical CVD, currently treated cancer or ever cancer with radiotherapy, and other serious medical conditions that would prevent long-term participation. The institutional review boards at all participating centers approved the study, and all participants provided signed informed consent. At the time of writing, the MESA cohort has provided longitudinal data from 7 follow-up visits spanning more than 20 years; this report includes the first 17.9 (IQR: 14.9-18.6) years of follow-up. Details of the design of the MESA longitudinal cohort have been previously described.^7^

### Inflammatory markers

Baseline blood samples were drawn on all MESA participants following a 12-hour fast, and ethylenediaminetetraacetic acid (EDTA)-anticoagulated plasma samples were stored at −70 °C. High-sensitivity C-reactive protein (hs-CRP) was measured using a BNII nephelometer (Dade Behring Inc). Intra-assay and interassay analytic coefficients of variation (CVs) ranged from 2.3% to 4.4% and from 2.1% to 5.7%, respectively. IL-6 was measured using an ultrasensitive ELISA (Quantikine HS Human IL-6 Immunoassay, R&D Systems). The laboratory analytic CV for this assay was 6.3%. D-dimer was measured using an immunoturbidimetric assay on a Sta-R analyzer (Liatest D-DI, Diagnostica Stago). The lower limit of detection was 0.01 μg/mL. GlycA is a composite biomarker of enzymatically glycosylated glycan N-acetylglucosamine residues on many of the most abundant acute-phase proteins in the serum, which has previously been described as a stable measure of low-grade, global inflammation.^8^ GlycA was measured using nuclear magnetic resonance LipoProfile spectra (CLIA-certified LipoScience, now Labcorp). CVs for intra-assay and interassay precision were 1.9% and 2.6%, respectively.

### ChrIRD and CVD events and death

Hospitalizations and deaths were identified by direct participant contact at 9-month to 12-month intervals. Deaths of participants who had emigrated or who dropped out of MESA were identified through the National Death Index.

The MESA database includes International Classification of Diseases (ICD)-coded conditions based on all hospitalizations and deaths. ChrIRD events and deaths were based on adjudication of these ICD diagnosis codes identified in participant hospitalization and death records. Only codes from discharge summaries and death summaries were included. Noncardiovascular, nondiabetes, and noncancer inflammatory-related diseases were identified by physician review of existing ICD rubrics, similar to previous studies.^9^, ^10^, ^11^ Conceptually, we considered non-CVD, nondiabetes, noncancer ChrIRD events to reflect a chronic inflammatory state produced by excess inflammation or infection. Adjudication of the ChrIRD variable was based on medical knowledge and was done as follows: 2 physicians independently reviewed the ICD codes and identified diagnoses reflecting noncardiovascular and noncancer conditions that had an important inflammatory component. These physicians had no information about the participant aside from each single ICD code, taken one at a time; multiple codes were not considered in each ChrIRD assignment. There was substantial initial agreement between the 2 reviewers, and a minimal subset of codes were discussed to obtain full agreement.

All ChrIRD codes were further stratified by severity, chronicity, primary organ system involved, and inflammatory physiology. Inflammatory physiology was empirically classified into 3 subgroups to separate inherently different clinical inflammatory processes. The subcategories were infectious pathologies, autoimmune or rheumatologic pathologies, and noninfectious nonautoimmune tissue damage pathologies. A representative sample of ChrIRD diagnosis codes, including the most frequently encountered conditions in this study, is summarized in Table 1.

**Table 1:** Sample of ChrIRD Code Categories and Associated Frequency by Number of Participants in the MESA Cohort

ICD Code Category	Total
Pneumonia	607
Sepsis	403
Abscess	231
Bronchitis	222
Gout	196
Cellulitis	194
COPD	172
Embolism	144
Pneumonitis	143
Hepatic	143
Renal	132
Diverticulitis	111
Biliary	94
Asthma	94
Rheumatoid arthritis	92
Appendicitis	75
Pancreatitis	66
Rhabdomyolysis	47
Influenza	45
Osteomyelitis	41
Emphysema	27
IBD	23
Other autoimmune^a^	60
Other hematologic^b^	37

Participants may be affected by more than 1 condition.

ChIRD = chronic inflammatory-related disease; COPD = chronic obstructive pulmonary disease; IBD = inflammatory bowel disease (ulcerative colitis or Crohn disease); ICD = International Classification of Diseases; MESA = Multi-Ethnic Study of Atherosclerosis.

a Other autoimmune category includes ankylosing spondylitis, antiphospholipid syndrome, autoimmune hemolytic anemias, celiac disease, dermatomyositis, diffuse diseases of connective tissue, Felty syndrome, giant cell arteritis, Goodpasture syndrome, granulomatosis with polyangiitis, heparin-induced thrombocytopenia, immune thrombocytopenic purpura, inclusion body myositis, inflammatory polyarthropathy, microscopic polyangiitis, polyarteritis nodosa, polymyositis, psoriatic arthropathy, and systemic sclerosis.

b Other hematologic category includes chronic myeloproliferative disease, disseminated intravascular coagulation, essential thrombocytopenia, monoclonal gammopathy, myelodysplastic syndrome, myelofibrosis, and polyclonal hypergammaglobulinemia.

The full list of ICD codes identified as ChrIRD is provided in Supplemental Table 1.

Per MESA protocol, a physician committee adjudicated CVD events based on medical records. CVD was defined as myocardial infarction, angina, stroke, transient ischemic attack, heart failure, peripheral arterial disease, and CVD death as previously described.^7^

### Statistical analysis

Mean ± SD or counts (percentages) of demographic factors and inflammatory biomarkers were calculated for description among 4 groups: participants without ChrIRD and CVD during the follow-up period, those with only a diagnosis of ChrIRD alone, those with only a diagnosis of CVD alone, and those with diagnoses of both ChrIRD and CVD. Differences in the inflammatory biomarkers among these 4 groups were assessed using linear regression, adjusted for age, race/ethnicity, and sex. The 3 degrees of freedom of the omnibus F-test for any differences among the 4 outcome categories was examined, and differences in inflammatory biomarkers among the 4 outcome groups were assessed as pairwise differences using SAS least squares means (pdiff option). Among those who suffered both ChrIRD and CVD diagnoses during follow-up, further breakdowns and cumulative mortality were described among those diagnosed with ChrIRD first, CVD first, and those with ChrIRD and CVD diagnosed in close proximity (within 30 days). ChrIRD diagnoses were further characterized according to acuteness of event, severity of event, and underlying general pathology and body system involved. We performed proportional hazard regression to identify future risk of either ChrIRD or CVD after time-dependent diagnosis of the alternate condition with adjustment for age, race/ethnicity, sex, and 4 biomarkers of inflammation (hs-CRP, IL-6, D-dimer, and GlycA). We similarly predicted total mortality according to time-dependent diagnosis of either ChrIRD or CVD, with the same baseline adjustments. Timing in the regression models was until the first occurrence of a given category of outcome (ChrIRD, CVD, or death) or censoring at last contact (mostly in 2019). Given only a small number of missing observations in the cohort, we used complete data analyses in these regressions. We note here that the 226 participants with any missing data have similar age and sex compared to the 6,565 with no missing data. The 226 were more likely to be Black race and less likely to be White race or Chinese ethnicity. Their cumulative event rates were slightly higher than in those with complete data: nominally higher for CVD (26% vs 21%, *P* > 0.05), but significantly higher for ChrIRD (37% vs 29%) and total death (37% vs 28%). A *P* value <0.05 was defined as statistical significance.

## Results

Participants (n = 6,791) had a mean age of 62 ± 10 years, with 47% (3,201/6,791) men, 39% (2,617/6,791) White, 28% (1,882/6,791) Black, 22% (1,489/6,791) Hispanic, and 12% (803/6,791) Chinese race/ethnicity (Table 2). Participants who suffered either ChrIRD or CVD were older and less likely to be Chinese. Males predominated in those with CVD, but there was no sex difference in those who suffered ChrIRD. Baseline inflammatory biomarkers tended to be higher in those who had either or both conditions during follow-up, with *P* value for the 3 degrees of freedom of F-test among the 4 outcome categories <0.00006 for each of the 4 biomarkers.

**Table 2:** Baseline Demographic and Inflammatory Biomarker Characteristics, MESA 2000-2002 (N = 6,790)

	Total		Neither CVD nor ChrIRD		CVD Only		ChrIRD Only		Both CVD and ChrIRD	
	N	% or Mean ± SD	N	% or Mean ± SD	N	% or Mean ± SD	N	% or Mean ± SD	N	% or Mean ± SD
Age (y)	6,791	62.1 ± 10.2	4,167	59.8 ± 9.8	667	65.3 ± 9.7	1,204	65 ± 10	753	67.9 ± 9.4
Male	3,201	47.1%	1,821	43.7%	407	61.0%	561	46.6%	412	54.7%
Race/ethnicity										
White	2,617	38.5%	1,528	36.7%	258	38.7%	514	42.7%	317	42.1%
Chinese	803	11.8%	574	13.8%	73	10.9%	102	8.5%	54	7.2%
Black	1,882	27.7%	1,128	27.1%	194	29.1%	346	28.7%	214	28.4%
Hispanic	1,489	21.9%	937	22.5%	142	21.3%	242	20.1%	168	22.3%
Biomarkers										
hsCRP (mg/L)	6,739	3.8 ± 5.8	4,138	3.4 ± 5.3	659	3.9 ± 6.2	1,192	4.5 ± 6.9	750	4.5 ± 6.5
IL-6 (pg/mL)	6,599	1.6 ± 1.2	4,068	1.4 ± 1.1	645	1.7 ± 1.3	1,158	1.8 ± 1.3	728	1.9 ± 1.4
D-dimer (ug/mL)	6,746	0.38 ± 0.87	4,139	0.31 ± 0.66	661	0.41 ± 1	1,195	0.46 ± 1.02	751	0.54 ± 1.33
GlycA (umol/L)	6,760	381.7 ± 62.2	4,150	377.1 ± 59.8	662	382.1 ± 61.5	1,198	388.5 ± 64.6	750	396.2 ± 68.7

Inflammatory biomarker variables: biomarker levels are baseline levels at study enrollment. Means within row with different letter superscripts are significantly different (*P* < 0.05) from each other in linear regression of each inflammatory biomarker (dependent variable) on the 4 disease categories, adjusted for age, race/ethnicity, and sex. A separate linear regression analysis was performed for each row. Comparing any 2 cells within a row, different superscripted letters indicate the 2 cells are significantly different (*P* < 0.05).

ChrIRD = chronic inflammatory-related disease; CVD = cardiovascular disease; hsCRP = high-sensitivity C-reactive protein; IL-6 = interleukin-6; MESA = Multi-Ethnic Study of Atherosclerosis.

A summary of the findings is shown in the Central Illustration. During the median 18-year follow-up, a diagnosis of ChrIRD occurred in 29% (1,965/6,791) of the cohort, and the cumulative incidence of CVD was 21% (1,420/6,791), including 11% (761/6,791) with both ChrIRD and CVD (Table 3). Among those diagnosed with both CVD and ChrIRD during the follow-up period, the median time from enrollment to first diagnosis of ChrIRD was 5.6 (Q1-Q3: 2.6-10.2) years and to incident CVD was 8.6 (Q1-Q3: 4.0-13.7) years. Among participants diagnosed with both ChrIRD and CVD during the follow-up period (n = 761), ChrIRD occurred first in 48% (368/761), CVD occurred first in 16% (121/761), and both diagnoses occurred within 30 days in 35% (264/761). Diagnosis sequence data are summarized in Table 4.

**Central Illustration:**
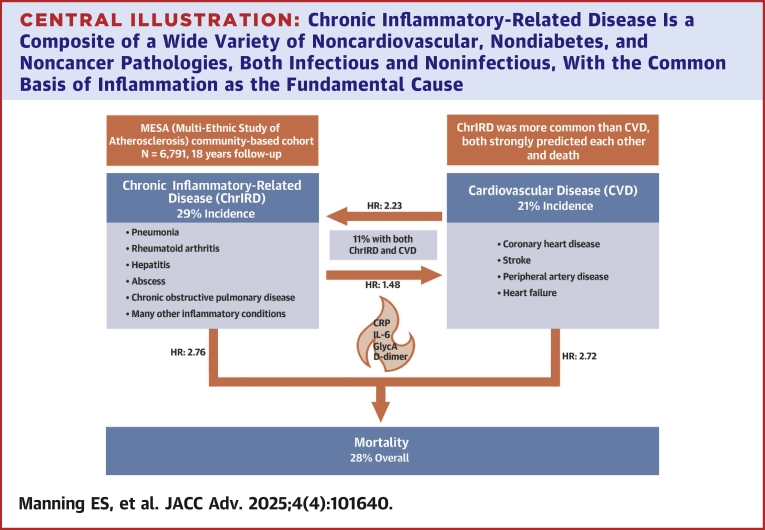
Chronic Inflammatory-Related Disease Is a Composite of a Wide Variety of Noncardiovascular, Nondiabetes, and Noncancer Pathologies, Both Infectious and Noninfectious, With the Common Basis of Inflammation as the Fundamental Cause ChrIRD was studied among 6,791 participants in the MESA (Multi-Ethnic Study of Atherosclerosis) over 18 years of follow-up. This clinical disease cluster is common, present in all organ systems, and is associated with high mortality, particularly in combination with cardiovascular disease (CVD). Our data suggest that these conditions share an underlying phenotype of inflammatory dysregulation and that the association between ChrIRD and CVD is bidirectional. ChIRD = chronic inflammatory-related disease.

**Table 3:** Joint Distribution, Time to Event, and Mortality According to New Diagnosis of CVD and ChrIRD

Condition	Total Diagnosed	All (N = 6,791)	Years to CVD	Years to ChrIRD	Deceased	Mortality
Neither	4,155	61.2	-	-	527	12.7
CVD only	659	9.7	8.6 (4-13.7)	-	300	45.5
ChrIRD only	1,204	17.8	-	5.6 (2.6-10.2)	567	47.1
Both CVD and ChrIRD	761	11.2	See Table 4	See Table 4	510	67

Values are n, %, or median (IQR).

ChrIRD = chronic inflammatory-related disease; CVD = cardiovascular disease.

**Table 4:** Sequencing of Occurrence Among People With Both Conditions

Condition	Total Diagnosed	Both (N = 761)	Years to CVD	Years to ChrIRD	Deceased	Mortality
ChrIRD first	368	48.9	9.3 (6.2-13.2)	3.0 (1.1-5.5)	250	67.9
CVD first	121	16.1	5.3 (1.5-12.5)	15.5 (9.6-18.2)	68	56.2
Same time	264	35.1	4.1 (2-8.7)	4.1 (2-8.7)	188	71.2

Values are n, %, or median (IQR).

ChrIRD = chronic inflammatory-related disease; CVD = cardiovascular disease.

Incident CVD was associated with an increased risk of future ChrIRD (HR: 1.48; 95% CI: 1.23-1.77) and a new diagnosis of ChrIRD was associated with an increased risk of future CVD (HR: 2.23; 95% CI: 1.97-2.52) after adjustment for age, race, and sex. Furthermore, in multivariable analysis after adjustment for age, race/ethnicity, and sex as well as for interim occurrence of the alternate condition, baseline hs-CRP, IL-6, D-dimer, and GlycA levels predicted future ChrIRD, and all inflammatory biomarkers except hs-CRP predicted future CVD (Table 5).

**Table 5:** Proportional Hazard Regressions Predicting CVD, ChrIRD, and Death During 18-Year Follow-Up (N = 6,565)

	CVD (n = 1,361)		ChrIRD (n = 1,889)		Death (n = 1,824)	
	HR (95% CI)	*P* Value	HR (95% CI)	*P* Value	HR (95% CI)	*P* Value
Time-dependent condition						
CVD	-	-	1.48 (1.23-1.77)	**<0.0001**	2.72 (2.45-3.01)	**<0.0001**
ChrIRD	2.23 (1.97-2.52)	**<0.0001**	-	-	2.76 (2.5-3.05)	**<0.0001**
Baseline biomarker levels						
IL-6	1.12 (1.07-1.18)	**<0.0001**	1.18 (1.13-1.23)	**<0.0001**	1.15 (1.1-1.2)	**<0.0001**
D-dimer	1.05 (1.01-1.09)	**0.009**	1.06 (1.03-1.1)	**0.0004**	1.08 (1.05-1.12)	**<0.0001**
GlycA	1.22 (1.14-1.29)	**<0.0001**	1.18 (1.12-1.24)	**<0.0001**	1.16 (1.1-1.22)	**<0.0001**

Each column represents a separate regression analysis, with baseline inflammatory biomarkers and time-dependent CVD and/or ChrIRD occurrence as predictors, adjusted for age, race/ethnicity, and sex. Inflammatory biomarkers are compared by SD.

IL-6-adjusted hsCRP is not shown because IL-6 is a precursor for hsCRP. **Bold** indicates *P* ≤ 0.009.

ChrIRD = chronic inflammatory-related disease; CVD = cardiovascular disease; hsCRP = high-sensitivity C-reactive protein; IL = interleukin.

The most common ChrIRD codes identified in the current analysis were pneumonia, sepsis, abscess, bronchitis, gout, cellulitis, and chronic obstructive lung disease (Table 1). The median (interquartile range) number of ChrIRD ICD codes identified during the follow-up period per participant with any ChrIRD diagnosis was 5 (2, 6) including a 95th percentile of 16 diagnoses. Participants could qualify for more than 1 ChrIRD subclassification. Nearly one-half of ChrIRD codes (46%, 915/1,977) were considered limited inflammatory conditions, that is, incident conditions at the time of diagnosis with expected resolution after treatment (such as acute infections). The most common ChrIRD events were infectious conditions (72%, 1,417/1,977), followed by conditions of noninfectious nonautoimmune tissue damage (58%; 1,156/1,977), and finally autoimmune/rheumatologic conditions (10%; 196/1,977). ChrIRD diagnoses were identified in all body systems but were most common in the pulmonary system and least common in the nonatherosclerotic cardiovascular system (Table 6).

**Table 6:** Organ and Body Systems Affected by ChrIRD Among 1,815 Participants With New Diagnoses of ChrIRD

System	Participants Affected	% of All Participants With Any ChrIRD Diagnosis	% of Participants Affected With Any Infectious ChrIRD Diagnosis	% of Participants Affected With ChrIRD Diagnosis in Additional Systems
Systemic	757	38.3	93.3	80.4
Nervous	57	2.9	80.7	75.4
Pulmonary	1,025	51.8	74.3	56.8
Cardiovascular^a^	40	2	77.5	82.5
Gastrointestinal	629	31.8	84.4	61.2
Renal	162	8.2	77.7	85.2
Musculoskeletal	298	15.1	63.1	71.4
Genitourinary	144	7.3	99.3	88.9
Skin	222	11.2	91.9	76.6
Ear nose throat	54	2.7	98.1	77.7
Endocrine^b^	66	3.3	51.5	69.7
Hematologic	50	2.5	68	72

ChrIRD = chronic inflammatory-related disease.

a Nonarteriosclerotic conditions such as endocarditis.

b Excludes diabetes.

Mortality among participants with neither CVD nor ChrIRD was 13% (527/4,155) during the median 18-year follow-up period. Mortality was higher among individuals with diagnosis of CVD but without ChrIRD (46%; 300/659), diagnosis of ChrIRD but without CVD (47%; 567/1,204), and highest among participants diagnosed with both conditions (67%; 510/761). Among 1,420 total CVD events, 32% (457/1,420) suffered death due to CVD and among 1,977 total ChrIRD events, 25% (488/1,977) suffered death due to ChrIRD. Mortality data are summarized in Tables 3 and 4. Both conditions and baseline inflammatory biomarkers predicted total death, after adjustment for age, race/ethnicity, and sex (Table 5).

## Discussion

Inflammatory dysregulation is complex, as it can remain subclinical and only be detected by elevated serum inflammatory markers or present clinically in any organ system with a wide range of minor to life-threatening diseases. The concept of ChrIRD, as presented in this paper and others, is an attempt to capture a composite of clinically meaningful manifestations of an underlying inflammatory process by leveraging clinically apparent diagnoses of inflammatory conditions via review of ICD codes.^9^, ^10^, ^11^ This composite simplifies the diverse phenotype of inflammatory disease into a clinically meaningful variable that focuses on the similarities of the hundreds of disparate component entities that form the basis of ChrIRD (Supplemental Table 1). As anticipated by its design and validated in these data, ChrIRD is associated with higher inflammatory biomarker levels years before the event and portends high mortality at or after the event. While the inflammatory mechanism and affected body system vary significantly across the cluster of ChrIRD conditions, there is a common association with increased CVD risk and increased mortality. This may indicate that ChrIRD, regardless of its final clinical phenotype, represents a common baseline propensity for inflammatory dysregulation that underpins its variable expression. Additionally, the association with CVD appears bidirectional: incident CVD increased the risk of future ChrIRD, and current ChrIRD increased the risk of future CVD.

The chronic inflammatory phenotype, which predisposes an individual to ChrIRD, may have a foundation in genetics, metabolism, the microbiome, and diet with eventual clinical penetrance in the form of ChrIRD. Genetic studies have implicated single-nucleotide polymorphisms in the IL-6 gene with increased inflammatory responses.^12^ Conditions such as the metabolic syndrome are known to increase systemic oxidative stress and reactive oxygen species.^13^ The immune system’s interaction with the gut microbiome can influence the development of autoimmune and inflammatory diseases.^14^ Studies from the Iowa Women’s Health Study and Coronary Artery Disease in Young Adults cohort have demonstrated that dietary patterns and overall diet quality are associated with both oxidative markers and inflammatory-related mortality.^15^^,^^16^ These inflammatory predispositions, some modifiable and others innate, are largely subclinical and attempts should be made to identify individuals at risk before the development of overt ChrIRD. This is supported by our analysis of baseline inflammatory markers, which predicted ChrIRD among participants at the time of enrollment when they were free of serious medical conditions that would prevent long-term participation.

These data also add to a growing body of evidence that inflammation plays an important role in the development and progression of CVD. Previous studies have demonstrated that individuals with a wide variety of chronic inflammatory and autoimmune conditions have an increased risk of CVD and that individuals with established CVD and elevated inflammatory markers are at higher risk of recurrent CVD events.^17^, ^18^, ^19^ In a Japanese study, Yaginuma et al studied 2,480 acute myocardial infarction patients undergoing percutaneous coronary interventions. They found substantial excess major adverse cardiovascular events and major bleeding events both during hospitalization and after hospitalization (median follow-up: 539 days) among patients with cancer or a selected set of chronic systemic inflammatory diseases compared to all other patients.^20^^,^^21^ Our data reiterate an association between inflammatory disease and CVD with an additional unique contribution of a bidirectional association.

The association between CVD and future inflammatory conditions in other organ systems is poorly understood. It has previously been described that, in the setting of acute myocardial infarction, circulating leukocytes form inflammatory networks that link the cardiovascular system to the central nervous system, autonomic nervous system, bone marrow, and spleen through cytokine release, leukocyte trafficking, and leukocyte production.^22^ It is possible that the immune dysregulation noted in these cardiovascular-mediated inflammatory networks could extend beyond the setting of acute myocardial infarction and explain the predisposition for future ChrIRD among individuals with diverse types of CVD. The local increase in oxidative stress from atherosclerotic plaques has been shown to alter the redox states of circulating leukocytes, which, in turn, may amplify and distribute further oxidative stress at the tissue level.^23^ Furthermore, cytokine and inflammasome gene expression in peripheral blood mononuclear cells, as determined by RNA sequencing, is similar between individuals with cardiomyopathy and those with COVID-19 infection.^24^ The specific impact of these inflammatory and immune-related changes in CVD on other organ systems, and particularly in relation to ChrIRD, requires further study.

The finding that ChrIRD is predictive of future CVD and that CVD is predictive of future ChrIRD suggests that the development of one of these inflammatory conditions could promote further baseline inflammatory dysregulation, which predisposes to additional inflammatory conditions. We interpret that these data reflect that an inflammatory condition is present in both ChrIRD and CVD, which underpins the predisposition to both conditions.

The clinical implications of these data, however, are less apparent. Our findings suggest that chronic, low-grade inflammation is a powerful risk factor for a wide range of severe diseases with high mortality. Clearly, the inclusion of a diagnosis in the composite of ChrIRD does not imply that there is a universal treatment for each component diagnostic entity. Many different clinical conditions are included in ChrIRD, which demand disease-specific treatment approaches. However, the utility of supplemental anti-inflammatory treatments for clinically apparent ChrIRD conditions requires further investigation. The most appropriate measures of chronic inflammation or targets for anti-inflammatory therapy for those at risk of future ChrIRD remain unknown. In this study, we evaluated 4 inflammatory biomarkers in relation to ChrIRD, but it is clear that any 1 or 2 biomarkers are insufficient to describe the complexity of an individual’s inflammatory profile and immune responsiveness. It is also uncertain if aggressive treatment or prevention of either subclinical or clinically apparent inflammation can reduce the risk of future ChrIRD or mortality. Control of excess inflammation using statin medications has demonstrated benefit in the secondary prevention of cardiovascular events.^25^ There have also been several trials exploring the use of other inflammation-modulating agents, including colchicine, methotrexate, and canakinumab, among individuals with coronary artery disease and elevated inflammatory biomarkers. Results have been mixed; however, most studies demonstrated at least a mild reduction in future cardiac events.^26^, ^27^, ^28^, ^29^, ^30^ Together, these trials highlight some clinical benefits that, in some cases, are offset by medication-related adverse events.

The importance of ChrIRD cannot be ignored and should inform future investigations. A better understanding of the inflammatory cascade in these chronic inflammatory conditions could provide novel therapeutic targets and improve patient selection for therapies based on biomarker profiles. Further exploration of ChrIRD conditions and the potential use of anti-inflammatory therapies may also provide insight into clinical management of these conditions.

### Study Limitations

This study is limited by its observational design. The data used to compile the ChrIRD variable may be limited somewhat by the level of completeness and accuracy of the collection of ICD codes from MESA participant hospital and death records. ChrIRD diagnoses in this study were limited to participants who were hospitalized or died during the study period.

## Conclusions

ChrIRD is common, is present in all organ systems, and is associated with high mortality, particularly in combination with CVD. Our data suggest that these conditions share an underlying phenotype of inflammatory dysregulation and are independent risk factors for each other. The association between CVD and ChrIRD is bidirectional, and future studies of the role of chronic inflammation in these conditions may produce new therapeutic targets and improved clinical management. The definition of ChrIRD proposed in this paper provides a framework for studying this important phenotype using observational data.

## Future directions

Considering the ongoing development of targeted anti-inflammatory agents and their expanding use in autoimmune and other inflammatory diseases, future studies should explore the effect of these agents on the development of both ChrIRD and CVD as well as primary and secondary cardiovascular events. The bidirectional nature of CVD and ChrIRD risk further suggests that these agents should be studied for the prevention of ChrIRD among individuals with established CVD.

## Funding support and author disclosures

This research was supported by contracts 75N92020D00001, HHSN268201500003I, N01-HC-95159, 75N92020D00005, N01-HC-95160, 75N92020D00002, N01-HC-95161, 75N92020D00003, N01-HC-95162, 75N92020D00006, N01-HC-95163, 75N92020D00004, N01-HC-95164, 75N92020D00007, N01-HC-95165, N01-HC-95166, N01-HC-95167, N01-HC-95168, and N01-HC-95169 from the 10.13039/100000050National Heart, Lung, and Blood Institute, and by grants UL1-TR-000040, UL1-TR-001079, and UL1-TR-001420 from the 10.13039/100006108National Center for Advancing Translational Sciences (NCATS). The authors have reported that they have no relationships relevant to the contents of this paper to disclose.
