# Supplementary material for: Chronic Inflammatory-Related Disease and Cardiovascular Disease in MESA
Source: JACC Adv. 2025 Mar 3;4(4):101640. doi: 10.1016/j.jacadv.2025.101640 (PMC11925088; doi:10.1016/j.jacadv.2025.101640)
Supplement: Supplemental Table 1 [file mmc1.pdf]

Supplementary Table 1: Diagnostic codes for ChrIRD (chronic inflammatory related disease) derived from International Classification of Diseases (ICD) versions 9 and 10.

ChrIRD subcategory	ICD rubric	ICD code	ICD version
Neurological Conditions	Jakob Creutzfeldt disease	46.1	9
	Unspecified viral meningitis	47.9	9
	Non-arthopod borne lymphocytic choriomeningitis	49	9
	Herpes zoster with unspecified nervous system complication	53.1	9
	Herpes zoster with other nervous system complications	53.19	9
	West Nile fever, unspecified	66.4	9
	Tabes dorsalis	94	9
	Neurosyphilis, unspecified	94.9	9
	Cysticercosis	123.1	9
	Pneumococcal meningitis	320.1	9
	Streptococcal meningitis	320.2	9
	Staphylococcal meningitis	320.3	9
	Meningitis due to gram-negative bacteria, not elsewhere classified	320.82	9
	Meningitis due to unspecified bacterium	320.9	9
	Cryptococcal meningitis	321	9
	Intracranial abscess	324	9
	Intraspinal abscess	324.1	9
	Multiple sclerosis	340	9
	Myasthenia gravis without (acute) exacerbation	358	9
	Myasthenia gravis	358	9
	Myasthenia gravis with (acute) exacerbation	358.01	9
	Cerebral arteritis	437.4	9
	Symptomatic neurosyphilis	A52.1	10
	Late syphilis: Symptomatic neurosyphilis	A52.1	10
	Atypical virus infections of central nervous system: Creutzfeldt-Jakob disea	A81.0	10
	Creutzfeldt-Jakob disease, unspecified	A81.00	10
	Atypical virus infections of central nervous system: Progressive multifocal l	A81.2	10
	Zoster ocular disease, unspecified	B02.30	10
	Meningitis, unspecified	G03.9	10
	Meningitis due to other and unspecified causes: Meningitis, unspecified	G03.9	10
	Amyotrophic lateral sclerosis	G12.21	10
	Multiple sclerosis	G35.	10
	Multiple sclerosis	G35.	10
	Guillain-Barre syndrome	G61.0	10
	Chronic inflammatory demyelinating polyneuritis	G61.81	10
	Myasthenia gravis	G70.0	10
	Myasthenia gravis and other myoneural disorders: Myasthenia gravis	G70.0	10
	Myasthenia gravis without (acute) exacerbation	G70.00	10
	Cerebral edema	G93.6	10
	Neurofibromatosis, unspecified	Q85.00	10
	Neurofibromatosis, type 1	Q85.01	10

	Unspecified pulmonary tuberculosis	11.9	9
	Unspecified pulmonary tuberculosis, confirmation unspecified	11.9	9
	Pulmonary diseases due to other mycobacteria	31	9
	Pulmonary actinomycotic infection	39.1	9
	Respiratory syncytial virus (RSV)	79.6	9
	Candidiasis of lung	112.4	9
	Histoplasma capsulatum pneumonia	115.05	9
	Aspergillosis	117.3	9
	Cryptococcosis	117.5	9
	Zygomycosis (phycomycosis or mucormycosis)	117.7	9
	Pneumocystosis	136.3	9
	Pulmonary embolism and infarction	415.1	9
	Iatrogenic pulmonary embolism and infarction	415.11	9
	Septic pulmonary embolism	415.12	9
	Saddle embolus of pulmonary artery	415.13	9
	Other pulmonary embolism and infarction	415.19	9
	Chronic pulmonary embolism	416.2	9
	Acute bronchitis and bronchiolitis	466	9
	Acute bronchitis	466	9
	Acute bronciolitis due to other infectious organisms	466.19	9
	Peritonsillar abscess	475	9
	Pneumonia due to parainfluenza virus	480.2	9
	Pneumonia due to other virus not elsewhere classified	480.8	9
	Viral pneumonia, unspecified	480.9	9
	Pneumococcal pneumonia [streptococcus pneumoniae pneumonia]	481	9
	Pneumonia due to klebsiella pneumoniae	482	9
	Pneumonia due to pseudomonas	482.1	9
	Pneumonia due to Hemophilus influenzae (h. Influenzae)	482.2	9
	Pneumonia due to streptococcus	482.3	9
	Pneumonia due to streptococcus, unspecified	482.3	9
	Pneumonia due to other streptococcus	482.39	9
	Pneumonia due to staphylococcus, unspecified	482.4	9
	Methicillin susceptible pneumonia due to staphylococcus aureus	482.41	9
	Methicillin resistant pneumonia due to staphylococcus aureus	482.42	9
	Pneumonia due to other specified bacteria	482.8	9
	Pneumonia due to anaerobes	482.81	9
	Pneumonia due to Escherichia coli [E.coli]	482.82	9
	Pneumonia due to other gram-negative bacteria	482.83	9
	Pneumonia due to legionnaires' disease	482.84	9
	Bacterial pneumonia, unspecified	482.9	9
	Pneumonia due to mycoplasma pneumoniae	483	9
	Pneumonia due to other specified organism	483.8	9
	Pneumonia in cytomegalic inclusion disease	484.1	9
	Pneumonia in anthrax	484.5	9

Bronchopneumonia, organism unspecified	485	9
Pneumonia, organism unspecified	486	9
Influenza with pneumonia	487	9
Influenza	487	9
Influenza with other respiratory manifestations	487.1	9
Influenza with other manifestations	487.8	9
Influenza due to identified novel h1n1 influenza virus	488.1	9
Influenza due to identified 2009 h1n1 influenza virus with other respiratory	488.12	9
Influenza due to identified novel influenza a virus with other respiratory ma	488.82	9
Bronchitis, not specified as acute or chronic	490	9
Chronic bronchitis	491	9
Simple chronic bronchitis	491	9
Obstructive chronic bronchitis	491.2	9
Obstructive chronic bronchitis, without exacerbation	491.2	9
Obstructive chronic bronchitis, with (acute) exacerbation	491.21	9
Obstructive chronic bronchitis with acute bronchitis	491.22	9
Other chronic bronchitis	491.8	9
Emphysema	492	9
Extrinsic asthma, with (acute) exacerbation	493.02	9
Chronic obstructive asthma with status asthmaticus	493.21	9
Chronic obstructive asthma, with (acute) exacerbation	493.22	9
Asthma, unspecified type, with (acute) exacerbation	493.92	9
Bronchiectasis without acute exacerbation	494	9
Bronchiectasis with acute exacerbation	494.1	9
Asbestosis	501	9
Pneumoconiosis due to other silica or silicates	502	9
Pneumonitis due to solids and liquids	507	9
Pneumonitis due to inhalation of food or vomitus	507	9
Acute pulmonary manifestations due to radiation	508	9
Empyema with fistula	510	9
Empyema without mention of fistula	510.9	9
Pleurisy with effusion, with mention of a bacterial cause other than tubercul	511.1	9
Malignant pleural effusion	511.81	9
Abscess of lung	513	9
Post inflammatory pulmonary fibrosis	515	9
Idiopathic fibrosing alveolitis	516.3	9
Idiopathic pulmonary fibrosis	516.31	9
Idiopathic non-specific interstitial pneumonitis	516.32	9
Other specified alveolar and parietoalveolar pneumonopathies	516.8	9
Interstitial emphysema	518.1	9
Pulmonary eosinophilia	518.3	9
Allergic bronchopulmonary aspergillosis	518.6	9
Rheumatoid lung	714.81	9
Ventilator associated pneumonia	997.31	9

**Pulmonary Conditions**	Tuberculosis of lung	A15.0	10
	Respiratory tuberculosis unspecified	A15.9	10
	Respiratory tuberculosis, not confirmed bacteriologically or histologically:	A16.9	10
	Pulmonary mycobacterial infection	A31.0	10
	Infection due to other mycobacteria: Pulmonary mycobacterial infection	A31.0	10
	Pneumocystosis	B59.	10
	Other enterovirus as the cause of diseases classified elsewhere	B97.19	10
	Respiratory syncytial virus as the cause of diseases classified elsewhere	B97.4	10
	Sarcoidosis of lung	D86.0	10
	Sarcoidosis: Sarcoidosis of lung	D86.0	10
	Inhalation and ingestion of food causing obstruction of respiratory tract or s	E911.	9
	Inhalation and ingestion of other object causing obstruction of respiratory tr	E912.	9
	Saddle embolus of pulmonary artery with acute cor pulmonale	I26.02	10
	Other pulmonary embolism with acute cor pulmonale	I26.09	10
	Pulmonary embolism: Pulmonary embolism without acute cor pulmonale	I26.9	10
	Saddle embolus of pulmonary artery without acute cor pulmonale	I26.92	10
	Other pulmonary embolism without acute cor pulmonale	I26.99	10
	Chronic pulmonary embolism	I27.82	10
	Acute upper respiratory infections of multiple and unspecified sites: Acute u	J06.9	10
	Influenza due to identified novel influenza a virus with other respiratory ma	J09.X2	10
	Influenza due to other identified influenza virus with unspecified type of pn	J10.00	10
	Influenza due to other identified influenza virus with other specified pneum	J10.08	10
	Influenza due to other identified influenza virus with other respiratory mani	J10.1	10
	Influenza due to unidentified influenza virus with other respiratory manifest	J11.1	10
	Human metapneumovirus pneumonia	J12.3	10
	Viral pneumonia, not elsewhere classified: Human metapneumovirus pneum	J12.3	10
	Viral pneumonia, unspecified	J12.9	10
	Pneumonia due to streptococcus pneumoniae	J13.	10
	Bacterial pneumonia, not elsewhere classified	J15.	10
	Pneumonia due to klebsiella pneumoniae	J15.0	10
	Pneumonia due to pseudomonas	J15.1	10
	Bacterial pneumonia, not elsewhere classified: Pneumonia due to Pseudomo	J15.1	10
	Bacterial pneumonia, not elsewhere classified: Pneumonia due to staphyloc	J15.2	10
	Bacterial pneumonia, not elsewhere classified: Pneumonia due to staphyloc	J15.21	10
	Pneumonia due to methicillin susceptible staphylococcus aureus	J15.211	10
	Pneumonia due to methicillin resistant staphylococcus aureus	J15.212	10
	Pneumonia due to other staphylococcus	J15.29	10
	Pneumonia due to other gram-negative bacteria	J15.6	10
	Pneumonia due to mycoplasma pneumoniae	J15.7	10
	Pneumonia due to other specified bacteria	J15.8	10
	Unspecified bacterial pneumonia	J15.9	10
	Pneumonia, unspecified organism	J18.	10
	Bronchopneumonia, unspecified organism	J18.0	10
	Pneumonia, organism unspecified: Bronchopneumonia, unspecified organis	J18.0	10

Lobar pneumonia, unspecified organism	J18.1	10
Pneumonia, organism unspecified: Lobar pneumonia, unspecified organism	J18.1	10
Pneumonia, organism unspecified: Hypostatic pneumonia, unspecified orga	J18.2	10
Pneumonia, unspecified organism	J18.9	10
Pneumonia, organism unspecified: Pneumonia, unspecified organism	J18.9	10
Acute bronchitis due to respiratory syncytial virus	J20.5	10
Acute bronchitis due to other specified organisms	J20.8	10
Acute bronchitis: Acute bronchitis, unspecified	J20.9	10
Acute bronchitis, unspecified	J20.9	10
Chronic sinusitis: Chronic sinusitis, unspecified	J32.9	10
Other diseases of upper respiratory tract: Other diseases of pharynx	J39.2	10
Bronchitis, not specified as acute or chronic	J40.	10
Unspecified chronic bronchitis	J42.	10
Unspecified chronic bronchitis	J42.	10
Centrilobular emphysema	J43.2	10
Other emphysema	J43.8	10
Emphysema, unspecified	J43.9	10
Emphysema: Emphysema, unspecified	J43.9	10
Other chronic obstructive pulmonary disease	J44.	10
Chronic obstructive pulmonary disease with (acute) lower respiratory infect	J44.0	10
Chronic obstructive pulmonary disease with acute lower respiratory infectio	J44.0	10
Other chronic obstructive pulmonary disease: Chronic obstructive pulmonar	J44.0	10
Chronic obstructive pulmonary disease with (acute) exacerbation	J44.1	10
Other chronic obstructive pulmonary disease: Chronic obstructive pulmonar	J44.9	10
Asthma	J45.	10
Mild intermittent asthma, uncomplicated	J45.20	10
Mild persistent asthma, uncomplicated	J45.30	10
Mild persistent asthma with (acute) exacerbation	J45.31	10
Moderate persistent asthma, uncomplicated	J45.40	10
Unspecified asthma with (acute) exacerbation	J45.901	10
Bronchiectasis with (acute) exacerbation	J47.1	10
Bronchiectasis, uncomplicated	J47.9	10
Pneumoconiosis due to asbestos and other mineral fibers	J61.	10
Pneumoconiosis due to dust containing silica: Pneumoconiosis due to other	J62.8	10
Pneumonitis due to inhalation of food and vomit	J69.0	10
Pneumonitis due to solids and liquids: Pneumonitis due to inhalation of foo	J69.0	10
Acute respiratory distress syndrome	J80.	10
Adult respiratory distress syndrome: Acute respiratory distress syndrome	J80.	10
Other interstitial pulmonary diseases	J84.	10
Other interstitial pulmonary diseases: Other interstitial pulmonary diseases	J84.1	10
Pulmonary fibrosis, unspecified	J84.10	10
Idiopathic pulmonary fibrosis	J84.112	10
Other interstitial pulmonary diseases: Interstitial pulmonary disease, unspec	J84.9	10
Abscess of lung and mediastinum: Gangrene and necrosis of lung	J85.0	10

	Abscess of lung with pneumonia	J85.1	10
	Infection of tracheostomy stoma	J95.02	10
	Transfusion-related acute lung injury (TRALI)	J95.84	10
	Rheumatoid lung disease with rheumatoid arthritis of unspecified site	M05.10	10
	Inhalation and ingestion of food causing obstruction of respiratory tract	W79.	10
	Inhalation and ingestion of other objects causing obstruction of respiratory t	W80.	10
**Non-atherosclerotic Cardiovascular Conditions**	Acute rheumatic pericarditis	391	9
	Acute pericarditis in diseases classified elsewhere	420	9
	Acute pericarditis, unspecified	420.9	9
	Acute idiopathic pericarditis	420.91	9
	Acute and subacute bacterial endocarditis	421	9
	Adhesive pericarditis	423.1	9
	Cardiac tamponade	423.3	9
	Endocarditis, valve unspecified	424.9	9
	Endocarditis, valve unspecified, unspecified cause	424.9	9
	Myocarditis, unspecified	429	9
	Dressler's syndrome	I24.1	10
	Other diseases of pericardium: Chronic constrictive pericarditis	I31.1	10
	Other diseases of pericardium: Pericardial effusion (noninflammatory)	I31.3	10
	Acute and subacute endocarditis	I33.	10
	Acute and subacute infective endocarditis	I33.0	10
	Acute and subacute endocarditis: Acute and subacute infective endocarditis	I33.0	10
	Endocarditis, valve unspecified	I38.	10
	Myocarditis, unspecified	I51.4	10
	Phlebitis and thrombophlebitis: Phlebitis and thrombophlebitis of other and	I80.2	10
	Cryptosporidiosis	7.4	9
	Intestinal infection due to e. Coli, unspecified	8	9
	Intestinal infection due to clostridium difficile	8.45	9
	Intestinal infection due to other anaerobes	8.46	9
	Intestinal infection due to other organism, not elsewhere classified	8.8	9
	Infectious colitis, enteritis, and gastroenteritis	9	9
	Colitis, enteritis, and gastroenteritis of presumed infectious origin	9.1	9
	Infectious diarrhea	9.2	9
	Diarrhea of presumed infectious origin	9.3	9
	Viral hepatitis	70	9
	Viral hepatitis a without mention of hepatic coma	70.1	9
	Viral hepatitis b with hepatic coma, acute or unspecified, without mention c	70.2	9
	Chronic viral hepatitis b with hepatic coma without hepatitis delta	70.22	9
	Viral hepatitis b without mention of hepatic coma	70.3	9
	Viral hepatitis b without mention of hepatic coma, acute or unspecified, wit	70.3	9
	Chronic viral hepatitis b without mention of hepatic coma without mention	70.32	9
	Chronic viral hepatitis b without mention of hepatic coma with hepatitis del	70.33	9
	Chronic hepatitis c with hepatic coma	70.44	9
	Acute hepatitis c without mention of hepatic coma	70.51	9

Hepatitis e without mention of hepatic coma	70.53	9
Chronic hepatitis c without mention of hepatic coma	70.54	9
Unspecified viral hepatitis with hepatic coma	70.6	9
Unspecified viral hepatitis c	70.7	9
Unspecified viral hepatitis c without hepatic coma	70.7	9
Unspecified viral hepatitis c with hepatic coma	70.71	9
Candidal esophagitis	112.84	9
Portal vein thrombosis	452	9
Budd-Chiari syndrome	453	9
Esophagitis	530.1	9
Acute esophagitis	530.12	9
Perforation of esophagus	530.4	9
Infection of gastrostomy	536.41	9
Acute appendicitis with generalized peritonitis	540	9
Acute appendicitis with peritoneal abscess	540.1	9
Acute appendicitis without mention of peritonitis	540.9	9
Appendicitis, unqualified	541	9
Incisional ventral hernia, with gangrene	551.21	9
Diaphragmatic hernia with gangrene	551.3	9
Hernia of other specified sites, with gangrene	551.8	9
Unilateral or unspecified femoral hernia with obstruction	552	9
Umbilical hernia with obstruction	552.1	9
Unspecified ventral hernia with obstruction	552.2	9
Incisional hernia with obstruction	552.21	9
Other ventral hernia with obstruction	552.29	9
Diaphragmatic hernia with obstruction	552.3	9
Regional enteritis of small intestine	555	9
Regional enteritis of small intestine with large intestine	555.2	9
Regional enteritis of unspecified site	555.9	9
Universal ulcerative (chronic) colitis	556.6	9
Ulcerative colitis, unspecified	556.9	9
Acute vascular insufficiency of intestine	557	9
Chronic vascular insufficiency of intestine	557.1	9
Eosinophilic gastroenteritis	558.41	9
Diverticulitis of small intestine (without mention of hemorrhage)	562.01	9
Diverticulitis of small intestine with hemorrhage	562.03	9
Diverticulitis of colon (without mention of hemorrhage)	562.11	9
Diverticulitis of colon with hemorrhage	562.13	9
Abscess of anal and rectal regions	566	9
Other suppurative peritonitis	567.2	9
Peritonitis (acute) generalized	567.21	9
Peritoneal abscess	567.22	9
Spontaneous bacterial peritonitis	567.23	9
Other suppurative peritonitis	567.29	9

Gastrointestinal Conditions	Other specified peritonitis	567.8	9
	Other specified peritonitis	567.89	9
	Abscess of intestine	569.5	9
	Acute and subacute necrosis of liver	570	9
	Acute alcoholic hepatitis	571.1	9
	Alcoholic cirrhosis of liver	571.2	9
	Chronic hepatitis, unspecified	571.4	9
	Autoimmune hepatitis	571.42	9
	Cirrhosis of liver without mention of alcohol	571.5	9
	Biliary cirrhosis	571.6	9
	Abscess of liver	572	9
	Hepatorenal syndrome	572.4	9
	Calculus of gallbladder with acute cholecystitis, with obstruction	574.01	9
	Calculus of gallbladder with other cholecystitis, with obstruction	574.11	9
	Calculus of bile duct with acute cholecystitis	574.3	9
	Calculus of bile duct with acute cholecystitis without mention of obstructio	574.3	9
	Calculus of bile duct with other cholecystitis, without mention of obstructio	574.4	9
	Calculus of bile duct with other cholecystitis, with obstruction	574.41	9
	Calculus of bile duct without mention of cholecystitis, with obstruction	574.51	9
	Calculus of gallbladder and bile duct with acute cholecystitis, without ment	574.6	9
	Calculus of gallbladder and bile duct with acute cholecystitis, with obstructi	574.61	9
	Calculus of gallbladder and bile duct with other cholecystitis, without menti	574.7	9
	Calculus of gallbladder and bile duct with other cholecystitis, with obstructi	574.71	9
	Calculus of gallbladder and bile duct with acute and chronic cholecystitis,	574.81	9
	Acute cholecystitis	575	9
	Chronic cholecystitis	575.11	9
	Acute and chronic cholecystitis	575.12	9
	Cholangitis	576.1	9
	Acute pancreatitis	577	9
	Chronic pancreatitis	577.1	9
	Celiac disease	579	9
	Chronic or unspecified parametritis and pelvic cellulitis	614.4	9
	Other intestinal Escherichia coli infections	A04.4	10
	Enterocolitis due to clostridium difficile	A04.7	10
	Other bacterial intestinal infections: Enterocolitis due to Clostridium diffici	A04.7	10
	Enterocolitis due to clostridium difficile, not specified as recurrent	A04.72	10
	Other specified bacterial intestinal infections	A04.8	10
	Other bacterial intestinal infections: Other specified bacterial intestinal infe	A04.8	10
	Viral intestinal infection, unspecified	A08.4	10
	Diarrhea and gastroenteritis of infectious origin	A09.	10
	Hepatitis a without hepatic coma	B15.9	10
	Acute hepatitis B without hepatic coma	B16.9	10
	Acute hepatitis B: Acute hepatitis B without delta-agent and without hepati	B16.9	10
	Acute hepatitis C	B17.1	10

Other acute viral hepatitis: Acute hepatitis C	B17.1	10
Chronic viral hepatitis b without delta-agent	B18.1	10
Chronic viral hepatitis c	B18.2	10
Chronic viral hepatitis: Chronic viral hepatitis C	B18.2	10
Unspecified viral hepatitis: Unspecified viral hepatitis without hepatic coma	B19.9	10
Candidal esophagitis	B37.81	10
Disorders of copper metabolism	E83.0	10
Portal vein thrombosis	I81.	10
Esophagitis	K20.	10
Perforation of esophagus	K22.3	10
Other diseases of esophagus: Perforation of esophagus	K22.3	10
Barrett's esophagus without dysplasia	K22.70	10
Acute appendicitis with generalized peritonitis	K35.2	10
Acute appendicitis with localized peritonitis	K35.3	10
Unspecified acute appendicitis	K35.80	10
Crohn's disease, unspecified, without complications	K50.90	10
Acute vascular disorders of intestine	K55.0	10
Vascular disorders of intestine: Acute vascular disorders of intestine	K55.0	10
Focal (segmental) acute infarction of small intestine	K55.021	10
Volvulus	K56.2	10
Diverticular disease of intestine: Diverticulitis of small intestine with perfor	K57.0	10
Diverticulitis of large intestine with perforation and abscess without bleedin	K57.20	10
Diverticulitis of intestine, part unspecified, with perforation and abscess wit	K57.80	10
Megacolon, not elsewhere classified	K59.3	10
Abscess of intestine	K63.0	10
Perforation of intestine (nontraumatic)	K63.1	10
Other diseases of intestine: Perforation of intestine (nontraumatic)	K63.1	10
Peritonitis: Peritonitis, unspecified	K65.9	10
Alcoholic cirrhosis of liver	K70.3	10
Alcoholic liver disease: Alcoholic cirrhosis of liver	K70.3	10
Alcoholic cirrhosis of liver without ascites	K70.30	10
Alcoholic liver disease: Alcoholic liver disease, unspecified	K70.9	10
Hepatic failure, not elsewhere classified: Acute and subacute hepatic failure	K72.0	10
Acute and subacute hepatic failure without coma	K72.00	10
Hepatic failure, unspecified	K72.9	10
Hepatic failure, not elsewhere classified: Hepatic failure, unspecified	K72.9	10
Hepatic failure, unspecified without coma	K72.90	10
Hepatic failure, not elsewhere classified: Hepatic failure, unspecified withou	K72.90	10
Chronic hepatitis, unspecified	K73.9	10
Primary biliary cirrhosis	K74.3	10
Fibrosis and cirrhosis of liver: Primary biliary cirrhosis	K74.3	10
Fibrosis and cirrhosis of liver: Other and unspecified cirrhosis of liver	K74.6	10
Abscess of liver	K75.0	10
Other inflammatory liver diseases: Abscess of liver	K75.0	10

	Autoimmune hepatitis	K75.4	10
	Other inflammatory liver diseases: Autoimmune hepatitis	K75.4	10
	Other inflammatory liver diseases: Inflammatory liver disease, unspecified	K75.9	10
	Other diseases of liver: Fatty (change of) liver, not elsewhere classified	K76.0	10
	Hepatorenal syndrome	K76.7	10
	Other diseases of liver: Hepatorenal syndrome	K76.7	10
	Calculus of gallbladder with acute cholecystitis	K80.0	10
	Calculus of gallbladder with acute cholecystitis without obstruction	K80.00	10
	Calculus of gallbladder with chronic cholecystitis without obstruction	K80.10	10
	Calculus of gallbladder with chronic cholecystitis with obstruction	K80.11	10
	Calculus of gallbladder with acute and chronic cholecystitis without obstruc	K80.12	10
	Calculus of bile duct with cholangitis, unspecified, without obstruction	K80.30	10
	Calculus of bile duct with acute cholangitis with obstruction	K80.33	10
	Calculus of bile duct with acute cholecystitis without obstruction	K80.42	10
	Calculus of bile duct with chronic cholecystitis with obstruction	K80.45	10
	Calculus of gallbladder and bile duct with acute cholecystitis with obstructi	K80.63	10
	Calculus of gallbladder and bile duct with chronic cholecystitis without obs	K80.64	10
	Acute cholecystitis	K81.0	10
	Cholecystitis: Acute cholecystitis	K81.0	10
	Chronic cholecystitis	K81.1	10
	Acute cholecystitis with chronic cholecystitis	K81.2	10
	Cholecystitis: Cholecystitis, unspecified	K81.9	10
	Perforation of gallbladder	K82.2	10
	Other diseases of gallbladder: Perforation of gallbladder	K82.2	10
	Cholangitis	K83.0	10
	Other diseases of biliary tract: Cholangitis	K83.0	10
	Obstruction of bile duct	K83.1	10
	Acute pancreatitis: Acute pancreatitis, unspecified	K85.9	10
	Acute pancreatitis without necrosis or infection, unspecified	K85.90	10
	Celiac disease	K90.0	10
	Pyogenic arthritis	711	9
	Pyogenic arthritis, site unspecified	711	9
	Pyogenic arthritis involving forearm	711.03	9
	Pyogenic arthritis involving pelvic region and thigh	711.05	9
	Pyogenic arthritis involving lower leg	711.06	9
	Pyogenic arthritis involving ankle and foot	711.07	9
	Unspecified infective arthritis involving lower leg	711.96	9
	Infective myositis	728	9
	Acute osteomyelitis, site unspecified	730	9
	Acute osteomyelitis involving lower leg	730.06	9
	Acute osteomyelitis involving ankle and foot	730.07	9
	Chronic osteomyelitis, site unspecified	730.1	9
	Chronic osteomyelitis involving pelvic region and thigh	730.15	9
	Chronic osteomyelitis involving ankle and foot	730.17	9

Musculoskeletal Conditions	Chronic osteomyelitis involving other specified sites	730.18	9
	Unspecified osteomyelitis, site unspecified	730.2	9
	Unspecified osteomyelitis involving shoulder region	730.21	9
	Unspecified osteomyelitis involving forearm	730.23	9
	Unspecified osteomyelitis involving hand	730.24	9
	Unspecified osteomyelitis involving pelvic region and thigh	730.25	9
	Unspecified osteomyelitis involving lower leg	730.26	9
	Unspecified osteomyelitis involving ankle and foot	730.27	9
	Unspecified osteomyelitis involving other specified sites	730.28	9
	Gangrene	785.4	9
	Lymphocutaneous sporotrichosis	B42.1	10
	Staphylococcal arthritis, left knee	M00.062	10
	Other streptococcal arthritis, left shoulder	M00.212	10
	Pyogenic arthritis: Pyogenic arthritis, unspecified	M00.9	10
	Other specified arthritis, left hip	M13.852	10
	Osteomyelitis of vertebra, thoracic region	M46.24	10
	Osteomyelitis of vertebra, lumbar region	M46.26	10
	Osteomyelitis of vertebra, sacral and sacrococcygeal region	M46.28	10
	Other chronic osteomyelitis, unspecified site	M86.60	10
	Other chronic osteomyelitis, left ankle and foot	M86.672	10
	Other osteomyelitis, other site	M86.8X8	10
	Osteomyelitis: Osteomyelitis, unspecified	M86.9	10
	Gangrene, not elsewhere classified	R02.	10
Genitourinary Conditions	Acute glomerulonephritis with unspecified pathological lesion in kidney	580.9	9
	Nephrotic syndrome with lesion of membranous glomerulonephritis	581.1	9
	Chronic glomerulonephritis with lesion of membranous glomerulonephritis	582.1	9
	Other chronic glomerulonephritis with specified pathological lesion in kidn	582.89	9
	Chronic glomerulonephritis with unspecified pathological lesion in kidney	582.9	9
	Nephritis and nephropathy, not specified as acute or chronic, in diseases cla	583.81	9
	End stage renal disease	585.6	9
	Chronic pyelonephritis without lesion of renal medullary necrosis	590	9
	Acute pyelonephritis	590.1	9
	Acute pyelonephritis	590.1	9
	Acute pyelonephritis without lesion of renal medullary necrosis	590.1	9
	Renal and perinephric abscess	590.2	9
	Chronic interstitial cystitis	595.1	9
	Other chronic cystitis	595.2	9
	Chronic prostatitis	601.1	9
	Other abscess of vulva	616.4	9
	Acute nephritic syndrome with unspecified morphologic changes	N00.9	10
	Acute nephritic syndrome: Acute nephritic syndrome with unspecified morp	N00.9	10
	Chronic nephritic syndrome with unspecified morphologic changes	N03.9	10
	Chronic nephritic syndrome: Chronic nephritic syndrome with unspecified	N03.9	10
	Nephrotic syndrome	N04.	10

	Nephrotic syndrome	N04.	10
	Unspecified nephritic syndrome with diffuse mesangiocapillary glomerulon	N05.5	10
	Acute pyelonephritis	N10.	10
	Chronic obstructive pyelonephritis	N11.1	10
	Tubulo-interstitial nephritis, not specified as acute or chronic	N12.	10
	Tubulo-interstitial nephritis, not specified as acute or chronic	N12.	10
	End stage renal disease	N18.6	10
	Acute cystitis without hematuria	N30.00	10
	Acute cystitis with hematuria	N30.01	10
	Interstitial cystitis (chronic) without hematuria	N30.10	10
	Cystitis, unspecified without hematuria	N30.90	10
	Urinary tract infection, site not specified	N39.0	10
	Other disorders of urinary system: Urinary tract infection, site not specified	N39.0	10
	Acute prostatitis	N41.0	10
	Congestion and hemorrhage of prostate	N42.1	10
**Dermatological Conditions**	Leprosy, unspecified	30.9	9
	Cellulitis and abscess of face	682	9
	Cellulitis and abscess of neck	682.1	9
	Cellulitis and abscess of trunk	682.2	9
	Cellulitis and abscess of upper arm and forearm	682.3	9
	Cellulitis and abscess of hand, except fingers and thumb	682.4	9
	Cellulitis and abscess of buttock	682.5	9
	Cellulitis and abscess of leg, except foot	682.6	9
	Cellulitis and abscess of foot, except toes	682.7	9
	Cellulitis and abscess of other specified sites	682.8	9
	Cellulitis and abscess of unspecified sites	682.9	9
	Acute lymphadenitis	683	9
	Cellulitis of right toe	L03.031	10
	Cellulitis of left toe	L03.032	10
	Cellulitis of unspecified toe	L03.039	10
	Cellulitis of left axilla	L03.112	10
	Cellulitis of right upper limb	L03.113	10
	Cellulitis of left upper limb	L03.114	10
	Cellulitis of right lower limb	L03.115	10
	Cellulitis of left lower limb	L03.116	10
	Cellulitis of neck	L03.221	10
	Cellulitis of abdominal wall	L03.311	10
	Cellulitis of chest wall	L03.313	10
	Cellulitis and acute lymphangitis, unspecified	L03.9	10
	Cellulitis: Cellulitis and acute lymphangitis, unspecified	L03.9	10
	Acute lymphadenitis: Acute lymphadenitis, unspecified	L04.9	10
	Pemphigus: Pemphigus, unspecified	L10.9	10
	Discoid lupus erythematosus	L93.0	10
	Lupus erythematosus: Discoid lupus erythematosus	L93.0	10

Non-Diabetes Endocrine Conditions	Thyrotoxicosis with or without goiter	242	9
	Thyrotoxicosis from ectopic thyroid nodule	242.4	9
	Thyrotoxicosis of other specified origin without mention of thyrotoxic crisis	242.8	9
	Thyrotoxicosis without mention of goiter or other cause	242.9	9
	Thyrotoxicosis without mention of goiter or other cause	242.9	9
	Thyrotoxicosis without mention of goiter or other cause, and without menti	242.9	9
	Chronic lymphocytic thyroiditis	245.2	9
	Thyroiditis, unspecified	245.9	9
	Diabetes with ketoacidosis	250.1	9
	Diabetes with ketoacidosis, type ii or unspecified type, not stated as uncontr	250.1	9
	Diabetes with ketoacidosis, type i [juvenile type], not stated as uncontrolled	250.11	9
	Diabetes with ketoacidosis, type ii or unspecified type, uncontrolled	250.12	9
	Diabetes with ketoacidosis, type i [juvenile type], uncontrolled	250.13	9
	Diabetes with hyperosmolarity	250.2	9
	Diabetes with hyperosmolarity, type ii or unspecified type, not stated as unc	250.2	9
	Diabetes with hyperosmolarity, type ii or unspecified type, uncontrolled	250.22	9
	Thyrotoxicosis with diffuse goiter without thyrotoxic crisis or storm	E05.00	10
	Thyrotoxicosis with toxic multinodular goiter without thyrotoxic crisis or st	E05.20	10
	Thyrotoxicosis, unspecified without thyrotoxic crisis or storm	E05.90	10
	Autoimmune thyroiditis	E06.3	10
	Insulin-dependent diabetes mellitus: Type 1 diabetes mellitus with ketoacid	E10.1	10
	Type 2 diabetes mellitus with hyperosmolarity without nonketotic hyperglyc	E11.00	10
	Type 2 diabetes mellitus with hyperosmolarity with coma	E11.01	10
	Primary adrenocortical insufficiency	E27.1	10
	Addisonian crisis	E27.2	10
Hematological Conditions	Amyloidosis	277.3	9
	Amyloidosis, unspecified	277.3	9
	Other amyloidosis	277.39	9
	Tumor lysis syndrome	277.88	9
	Autoimmune hemolytic anemias	283	9
	Immune thrombocytopenic purpura	287.31	9
	Heparin-induced thrombocytopenia (hit)	289.84	9
	Unspecified human immunodeficiency virus [HIV] disease	B24.	10
	Myelodysplastic syndromes	D46.	10
	Myelodysplastic syndrome, unspecified	D46.9	10
	Myelodysplastic syndromes: Myelodysplastic syndrome, unspecified	D46.9	10
	Chronic myeloproliferative disease	D47.1	10
	Monoclonal gammopathy	D47.2	10
	Essential (hemorrhagic) thrombocythemia	D47.3	10
	Disseminated intravascular coagulation [defibrination syndrome]	D65.	10
	Antiphospholipid syndrome	D68.61	10
	Immune thrombocytopenic purpura	D69.3	10
	Monocytosis (symptomatic)	D72.821	10
	Myelofibrosis	D75.81	10

	Heparin induced thrombocytopenia (hit)	D75.82	10
	Certain diseases involving lymphoreticular tissue and reticulohistiocytic sys	D76.2	10
	Polyclonal hypergammaglobulinemia	D89.0	10
	Organ-limited amyloidosis	E85.4	10
	Amyloidosis, unspecified	E85.9	10
	Amyloidosis: Amyloidosis, unspecified	E85.9	10
	Sarcoidosis	135	9
	Gouty arthropathy, unspecified	274	9
	<Invalid code effective 10/2009> gouty arthropathy	274	9
	Gouty arthropathy, unspecified	274	9
	Acute gouty arthropathy	274.01	9
	Chronic gouty arthropathy with tophus (tophi)	274.03	9
	Gout with other specified manifestations	274.89	9
	Gout, unspecified	274.9	9
	Inclusion body myositis	359.71	9
	Polyarteritis nodosa	446	9
	Goodpasture's syndrome	446.21	9
	Wegener's granulomatosis	446.4	9
	Giant cell arteritis	446.5	9
	Inflammatory conditions of jaw	526.4	9
	Stevens-johnson syndrome	695.13	9
	Lupus erythematosus	695.4	9
	Psoriatic arthropathy	696	9
	Diffuse diseases of connective tissue	710	9
	Systemic lupus erythematosus	710	9
	Systemic sclerosis	710.1	9
	Sicca syndrome	710.2	9
	Dermatomyositis	710.3	9
	Polymyositis	710.4	9
	Other specified diffuse diseases of connective tissue	710.8	9
	Rheumatoid arthritis and other inflammatory polyarthropathies	714	9
	Rheumatoid arthritis	714	9
	Felty's syndrome	714.1	9
	Other rheumatoid arthritis with visceral or systemic involvement	714.2	9
	Chronic or unspecified polyarticular juvenile rheumatoid arthritis	714.3	9
	Ankylosing spondylitis	720	9
	Polymyalgia rheumatica	725	9
**Rheumatological Conditions**	Rhabdomyolysis	728.88	9
	Disseminated intravascular coagulation [defibrination syndrome]	D65.	10
	Sarcoidosis of other sites	D86.89	10
	Sarcoidosis: Sarcoidosis, unspecified	D86.9	10
	Hereditary hemochromatosis	E83.110	10
	Rheumatoid arthritis with rheumatoid factor, unspecified	M05.9	10
	Inflammatory polyarthropathy	M06.4	10

	Other specified rheumatoid arthritis	M06.8	10
	Other specified rheumatoid arthritis, unspecified site	M06.80	10
	Rheumatoid arthritis, unspecified	M06.9	10
	Other rheumatoid arthritis: Rheumatoid arthritis, unspecified	M06.9	10
	Idiopathic gout, unspecified site	M10.00	10
	Idiopathic gout, left knee	M10.062	10
	Idiopathic gout, right ankle and foot	M10.071	10
	Idiopathic gout, multiple sites	M10.09	10
	Gout, unspecified	M10.9	10
	Gout: Gout, unspecified	M10.9	10
	Chronic gout, unspecified	M1A.9	10
	Chronic gout, unspecified, without tophus (tophi)	M1A.9XX0	10
	Chronic gout, unspecified, with tophus (tophi)	M1A.9XX1	10
	Other necrotizing vasculopathies: Hypersensitivity angiitis	M31.0	10
	Other giant cell arteritis	M31.6	10
	Microscopic polyangiitis	M31.7	10
	Other specified necrotizing vasculopathies	M31.8	10
	Systemic lupus erythematosus, unspecified	M32.9	10
	Other systemic involvement of connective tissue: Sicca syndrome [Sjogren]	M35.0	10
	Sjogren syndrome	M35.0	10
	Other systemic involvement of connective tissue: Polymyalgia rheumatica	M35.3	10
	Polymyalgia rheumatica	M35.3	10
	Ankylosing spondylitis lumbar region	M45.6	10
	Other inflammatory spondylopathies	M46.	10
	Rhabdomyolysis	M62.82	10
	Other osteonecrosis, left femur	M87.852	10
**Head and Neck Conditions**	Streptococcal sore throat	34	9
	Acute suppurative otitis media with spontaneous rupture of eardrum	382.01	9
	Acute maxillary sinusitis	461	9
	Acute sphenoidal sinusitis	461.3	9
	Acute sinusitis, unspecified	461.9	9
	Acute pharyngitis	462	9
	Acute tonsillitis	463	9
	Acute laryngitis without mention of obstruction	464	9
	Acute laryngotracheitis without mention of obstruction	464.2	9
	Supraglottitis unspecified without obstruction	464.5	9
	Cellulitis of pharynx or nasopharynx	478.21	9
	Dental caries, unspecified	521	9
	Acute apical periodontitis of pulpal origin	522.4	9
	Periapical abscess without sinus	522.5	9
	Aggressive periodontitis, unspecified	523.3	9
	Cellulitis and abscess of oral soft tissues	528.3	9
	Acute frontal sinusitis, unspecified	J01.10	10
	Acute pharyngitis, unspecified	J02.9	10

	Acute laryngitis	J04.0	10
Systemic Conditions	Disseminated mycobacterium	31.2	9
	Streptococcal septicemia	38	9
	Staphylococcal septicemia, unspecified	38.1	9
	Methicillin susceptible staphylococcus aureus septicemia	38.11	9
	Methicillin resistant staphylococcus aureus septicemia	38.12	9
	Other staphylococcal septicemia	38.19	9
	Pneumococcal septicemia	38.2	9
	Septicemia due to anaerobes	38.3	9
	Septicemia due to gram-negative organism, unspecified	38.4	9
	Septicemia due to Escherichia coli (e. Coli)	38.42	9
	Septicemia due to pseudomonas	38.43	9
	Other septicemia due to gram-negative organisms	38.49	9
	Other specified septicemias	38.8	9
	Unspecified septicemia	38.9	9
	Actinomycotic infection of unspecified site	39.9	9
	Gas gangrene	40	9
	Klebsiella pneumoniae	41.3	9
	Other and unspecified Escherichia coli [e. Coli]	41.49	9
	Helicobacter pylori [h. Pylori]	41.86	9
	Human immunodeficiency virus (hiv) disease	42	9
	Cytomegaloviral disease	78.5	9
	Human immunodeficiency virus, type 2 [hiv-2]	79.53	9
	Mixed malaria	84.5	9
	Lyme disease	88.81	9
	Syphilis, unspecified	97.9	9
	Lymphangitis	457.2	9
	Infection due to central venous catheter	999.31	9
	Bloodstream infection due to central venous catheter	999.32	9
	Septicemia due to streptococcus, group b	A40.1	10
	Septicemia due to streptococcus pneumoniae	A40.3	10
	Sepsis due to staphylococcus aureus	A41.0	10
	Other septicemia: Sepsis due to Staphylococcus aureus	A41.0	10
	Sepsis due to methicillin susceptible staphylococcus aureus	A41.01	10
	Sepsis due to methicillin resistant staphylococcus aureus	A41.02	10
	Sepsis due to unspecified staphylococcus	A41.2	10
	Other septicemia: Sepsis due to unspecified staphylococcus	A41.2	10
	Septicemia due to Hemophilus influenzae	A41.3	10
	Other septicemia: Sepsis due to other Gram-negative organisms	A41.5	10
	Gram-negative sepsis, unspecified	A41.50	10
	Sepsis due to Escherichia coli [e. Coli]	A41.51	10
	Other gram-negative sepsis	A41.59	10
	Other septicemia: Other specified sepsis	A41.8	10
	Sepsis due to enterococcus	A41.81	10

Septicemia, unspecified	A41.9	10
Other septicemia: Sepsis, unspecified organism	A41.9	10
Actinomycosis: Actinomycotic sepsis	A42.7	10
Actinomycosis: Actinomycosis, unspecified	A42.9	10
Other forms of nocardiosis	A43.8	10
Legionnaires' disease	A48.1	10
Other bacterial diseases, not elsewhere classified: Legionnaires' disease	A48.1	10
Bacterial infection of unspecified site: Staphylococcal infection, unspecified	A49.0	10
Bacterial infection of unspecified site: Other bacterial infections of unspecif	A49.8	10
Bacterial infection of unspecified site: Bacterial infection, unspecified	A49.9	10
Human immunodeficiency virus [hiv] disease	B20.	10
Human immunodeficiency virus [HIV] disease resulting in infectious and pa	B20.	10
HIV disease resulting in other specified conditions	B23.8	10
Human immunodeficiency virus [HIV] disease resulting in other conditions	B23.8	10
Unspecified human immunodeficiency virus disease	B24.	10
Cytomegaloviral disease: Other cytomegaloviral diseases	B25.8	10
Candidal septicemia	B37.7	10
Candidiasis: Candidiasis, unspecified	B37.9	10
Paracoccidioidomycosis, unspecified	B41.9	10
Sequelae of other and unspecified infectious and parasitic diseases: Sequela	B94.8	10
Streptococcus, group b, as the cause of diseases classified elsewhere	B95.1	10
Enterococcus as the cause of diseases classified elsewhere	B95.2	10
Other streptococcus as the cause of diseases classified elsewhere	B95.4	10
Methicillin susceptible staphylococcus aureus infection as the cause of disea	B95.61	10
Methicillin resistant staphylococcus aureus infection as the cause of disease	B95.62	10
Other staphylococcus as the cause of diseases classified elsewhere	B95.7	10
Unspecified staphylococcus as the cause of diseases classified elsewhere	B95.8	10
Klebsiella pneumoniae [k. Pneumoniae] as the cause of diseases classified e	B96.1	10
Escherichia coli [e. Coli] as the cause of diseases classified elsewhere	B96.2	10
Unspecified Escherichia coli [e. Coli] as the cause of diseases classified els	B96.20	10
Proteus (mirabilis) (morganii) as the cause of diseases classified elsewhere	B96.4	10
Pseudomonas (aeruginosa) (mallei) (pseudomallei) as the cause of diseases	B96.5	10
Bacteroides fragilis [b. Fragilis] as the cause of diseases classified elsewhere	B96.6	10
Other specified bacterial agents as the cause of diseases classified elsewhere	B96.89	10
Lymphangitis	I89.1	10
Bacteremia	R78.81	10
Bloodstream infection due to central venous catheter, initial encounter	T80.211A	10
Infection following a procedure	T81.4	10
Streptococcus infection in conditions classified elsewhere and of unspecifie	41	9
Streptococcus infection in conditions classified elsewhere and of unspecifie	41	9
Streptococcus infection in conditions classified elsewhere and of unspecifie	41.01	9
Streptococcus infection in conditions classified elsewhere and of unspecifie	41.02	9
Streptococcus infection in conditions classified elsewhere and of unspecifie	41.04	9
Streptococcus infection in conditions classified elsewhere and of unspecifie	41.05	9

**Infectious Conditions Not Otherwise Specified**	Streptococcus infection in conditions classified elsewhere and of unspecifie	41.09	9
	Staphylococcus infection in conditions classified elsewhere and of unspecif	41.1	9
	Staphylococcus infection in conditions classified elsewhere and of unspecif	41.1	9
	Methicillin susceptible staphylococcus aureus in conditions classified elsew	41.11	9
	Methicillin resistant staphylococcus aureus in conditions classified elsewhe	41.12	9
	Staphylococcus infection in conditions classified elsewhere and of unspecif	41.19	9
	Escherichia coli (e. Coli) infection in conditions classified elsewhere and of	41.4	9
	Hemophilus influenzae (h. Influenzae) infection in conditions classified els	41.5	9
	Proteus (mirabilis) (morganii) infection in conditions classified elsewhere a	41.6	9
	Pseudomonas infection in conditions classified elsewhere and of unspecifie	41.7	9
	Other specified bacterial infections in conditions classified elsewhere and o	41.83	9
	Other specified bacterial infections in conditions classified elsewhere and o	41.84	9
	Other specified bacterial infections in conditions classified elsewhere and o	41.85	9
	Other specified bacterial infections in conditions classified elsewhere and o	41.89	9
	Bacterial infection, unspecified, in conditions classified elsewhere and of un	41.9	9
